# The Polymorphism at PLCB4 Promoter (rs6086746) Changes the Binding Affinity of RUNX2 and Affects Osteoporosis Susceptibility: An Analysis of Bioinformatics-Based Case-Control Study and Functional Validation

**DOI:** 10.3389/fendo.2021.730686

**Published:** 2021-11-25

**Authors:** Dung-Jang Tsai, Wen-Hui Fang, Li-Wei Wu, Ming-Cheng Tai, Chung-Cheng Kao, Shih-Ming Huang, Wei-Teing Chen, Po-Jen Hsiao, Chih-Chien Chiu, Wen Su, Chia-Chun Wu, Sui-Lung Su

**Affiliations:** ^1^ Graduate Institute of Life Sciences, National Defense Medical Center, Taipei, Taiwan; ^2^ School of Public Health, National Defense Medical Center, Taipei, Taiwan; ^3^ Department of Family and Community Medicine, Tri-Service General Hospital, National Defense Medical Center, Taipei, Taiwan; ^4^ Department of Ophthalmology, Tri-Service General Hospital, National Defense Medical Center, Taipei, Taiwan; ^5^ Superintendent’s Office, Tri-Service General Hospital Songshan Branch, National Defense Medical Center, Taipei, Taiwan; ^6^ Department of Biochemistry, National Defense Medical Center, Taipei, Taiwan; ^7^ Division of Thoracic Medicine, Department of Medicine, Cheng Hsin General Hospital, Taipei, Taiwan; ^8^ Department of Medicine, Tri-Service General Hospital, National Defense Medical Center, Taipei, ROC, Taiwan; ^9^ Department of Internal Medicine, Taoyuan Armed Forces General Hospital, Taoyuan, Taiwan; ^10^ Division of Nephrology, Department of Internal Medicine, Tri-Service General Hospital, National Defense Medical Center, Taipei, Taiwan; ^11^ Division of Infectious Diseases, Department of Internal Medicine, Taoyuan Armed Forces General Hospital, National Defense Medical Center, Taoyuan, Taiwan; ^12^ Graduate Institute of Aerospace and Undersea Medicine, National Defense Medical Center, Taipei, Taiwan; ^13^ Department of Orthopedics, Tri-Service General Hospital, National Defense Medical Center, Taipei, Taiwan

**Keywords:** osteoporosis, runt domain transcription factor 2, binding site polymorphism, case-control study, PLCB4

## Abstract

**Purpose:**

Genome-wide association studies have identified numerous genetic variants that are associated with osteoporosis risk; however, most of them are present in the non-coding regions of the genome and the functional mechanisms are unknown. In this study, we aimed to investigate the potential variation in runt domain transcription factor 2 (RUNX2), which is an osteoblast-specific transcription factor that normally stimulates bone formation and osteoblast differentiation, regarding variants within RUNX2 binding sites and risk of osteoporosis in postmenopausal osteoporosis (PMOP).

**Methods:**

We performed bioinformatics-based prediction by combining whole genome sequencing and chromatin immunoprecipitation sequencing to screen functional SNPs in the RUNX2 binding site using data from the database of Taiwan Biobank; Case-control studies with 651 postmenopausal women comprising 107 osteoporosis patients, 290 osteopenia patients, and 254 controls at Tri-Service General Hospital between 2015 and 2019 were included. The subjects were examined for bone mass density and classified into normal and those with osteopenia or osteoporosis by T-scoring with dual-energy X-ray absorptiometry. Furthermore, mRNA expression and luciferase reporter assay were used to provide additional evidence regarding the associations identified in the association analyses. Chi-square tests and logistic regression were mainly used for statistical assessment.

**Results:**

Through candidate gene approaches, 3 SNPs in the RUNX2 binding site were selected. A novel SNP rs6086746 in the PLCB4 promoter was identified to be associated with osteoporosis in Chinese populations. Patients with AA allele had higher risk of osteoporosis than those with GG+AG (adjusted OR = 6.89; 95% confidence intervals: 2.23–21.31, p = 0.001). Moreover, the AA genotype exhibited lower bone mass density (p < 0.05). Regarding mRNA expression, there were large differences in the correlation between PLCB4 and different RUNX2 alleles (Cohen’s q = 0.91). Functionally, the rs6086746 A allele reduces the RUNX2 binding affinity, thus enhancing the suppression of PLCB4 expression (p < 0.05).

**Conclusions:**

Our results provide further evidence to support the important role of the SNP rs6086746 in the etiology of osteopenia/osteoporosis, thereby enhancing the current understanding of the susceptibility to osteoporosis. We further studied the mechanism underlying osteoporosis regulation by PLCB4.

## 1 Introduction

Osteoporosis is a systemic bone disease and is characterized by significant decrease in bone mass density (BMD) and damage to bone microstructure (1). This is especially noted in postmenopausal women because the prevalence of osteopenia and osteoporosis increase with age (2). Researchers estimate that there are >200 million osteoporosis patients globally and the risk of fracture in osteoporosis patients is as high as 40% (3, 4). To make the matter worse, the number of osteoporosis patients is expected to increase continuously owing to the effects of global population aging (5). A Taiwanese survey showed that osteoporosis ranks 4th among chronic diseases in elderly people aged >65 years in Taiwan and its prevalence is increasing with population aging (6). In addition, there are 12.3% of adults aged >50 years with at least one site with osteoporosis (the T-score of at least one vertebra or femur ≤ −2.5). Regarding sex, 8.6% and 15.5% of males and females, respectively, have one site with osteoporosis (7).

Genetic and environmental factors may affect osteoporosis progression (8). In addition to aging and other environmental factors, genetics is also an important factor that determines BMD (9). Osteoporosis is considered to be the outcome of interactions among several gene mutations (10). The results of past studies on twins and family data estimated that approximately 50%–85% of osteoporosis causes can be attributed to genetic factors (4, 11). In clinical practice, BMD is an important marker of osteoporosis and is a key marker for the diagnosis and treatment of osteoporosis (12). Therefore, there is a need to comprehensively understand the genetic factors involved in osteoporosis and BMD for the development of effective treatments for osteoporosis. In recent years, with the development of microarray and next-generation sequencing, genome-wide association studies (GWAS) is considered a valuable tool for studying complex genetic diseases. Since 2007, GWAS has confirmed several hundred susceptible loci for osteoporosis and BMD (12–15). However, most genome-wide significant susceptibility loci are located in non-coding regions in the genome and can provide only limited information on the genetic mechanisms of osteoporosis (16, 17). One of the primary molecular mechanisms by which SNPs regulate disease susceptibility is affecting the transcription factor binding, thereby regulating gene expression (18). Among these regions, transcription factor binding sites (TFBSs) on DNA play a central role in gene regulation *via* their sequence-specific interactions with transcription factor proteins (19).

With the recent progress in osteoporosis-related studies, we understood that the effects of osteoclasts and osteoblasts result in an imbalance between bone destruction and formation, which ultimately causes a decrease in bone mass and bone mineral density (20, 21). RUNX2 is one of the most important transcription factors and is also a key transcription regulatory factor in osteoblast differentiation. Therefore, it plays an important role in regulating osteoblast maturation and balance (22, 23). Recent studies showed that RUNX2 expression and BMD are positively correlated (24–28). During the differentiation of mesenchymal stem cells, RUNX2 regulates the gene transcription of key proteins and aid in the cells’ differentiation into osteoblasts (29).

Therefore, the aim of this study was to examine the correlation between the potential DNA binding sites of RUNX2 and osteoporosis. Considering the abovementioned facts, we used a bioinformatics-based approach to identify SNPs within osteoporosis-associated TFBSs. These genetic variations, which may directly affect post-transcriptional regulation of gene expression of transcription factors through SNPs present in the protein sequence, were assessed with respect to their potential association with osteoporosis susceptibility.

## 2 Materials and Methods

### 2.1 Study Participants

This hospital-based case-control study was conducted between March 2015 and October 2019. In the study cohort, 107 patients with osteoporosis, 290 patients with osteopenia, and 254 healthy controls were enrolled from Tri-Service General Hospital. All subjects included in the study were randomly chosen and excluded osteoporosis patients with ICD-10 M81.8 after consulting the medical records by the orthopedist. The BMD of all subjects was measured using dual-energy X-ray absorptiometry (DEXA) at the lumbar spine (LS1-4), and the diagnosis of osteoporosis was based on the World Health Organization standards. None of the subjects had a history of medication for osteoporosis treatment. The demographic and clinical characteristics of all subjects were obtained from questionnaires and medical records.

### 2.2 Bone Marrow Density Measurements

BMD (g/cm2) is used as an indicator of osteoporosis and is calculate by dividing the bone mineral content (g) by bone area (cm^2^) (30). In our study, BMD were measured by DEXA during health examinations at TSGH using Prodigy Series X-Ray Tube Housing Assembly (GE Medical Systems Lunar 3030 Ohmeda Dr Madison, Wisconsin, USA) (31). Osteoporosis was defined according to World Health Organization criteria that considers BMD measurements at or below −2.5 standard deviation (S.D.) from the optimal peak bone density (T-score) of healthy young adult of the same sex; conversely, BMD measurement at or above −1 S.D. from the optimal peak bone density of healthy young adult of the same sex was considered bone mass loss or normal (32).

### 2.3 Process of Bioinformatics Analysis of Candidate SNPs in TFBSs

#### 2.3.1 Screen the Genetic Variation in the Genome of Taiwanese Through Quality Control Procedures

First, we used the next-generation sequencing (NGS) data of 1,517 people released by the Taiwan Biobank, which contains 74,861,556 genetic variants. We deleted structural variants (insertion/deletion) because there was no way to use the multifunctional mass spectrometer (mass array) for genotyping. Then, we excluded SNPs with call rate of <90%. Finally, the remaining SNPs were used for further alignment.

#### 2.3.2 Identify Genetic Variants That May Affect RUNX2 Binding Motif Through Bioinformatics Sequence Alignment

Second, we analyzed genetic variants that may affect RUNX2 binding by using bioinformatics sequence alignment techniques and identified the variants located in the TFBS. In the past, the TFBS sequence of the identified transcription factor was 5’-HGHGGK-3’ (H = A, C or T; K = G or T). We aligned this motif found 1,672,016 SNPs that may affect the binding affinity.

#### 2.3.3 Chromatin Immunoprecipitation Sequencing Confirms That These Genetic Variants Bind to These Binding Motifs

Third, we used ChIP-Seq data to verify whether these SNPs combine with RUNX2 in the chromatin immunoprecipitation experiment. The study was performed using SAOS-2 cells for ChIP-Seq analysis and analysis of the RUNX2 proteins of TFBS and was published in the JASPAR database (Matrix ID: MA0511.1) (33). Based on 1,062 motifs of RUNX2 Chip-Seq data, three SNPs that affect RUNX2 binding affinity were filtered out.

### 2.4 Genomic DNA Extraction and SNP Genotyping

Blood samples were obtained from all subjects in the morning while they were in a fasting state. Genomic DNA was isolated from peripheral blood samples using standard procedures for proteinase K (Invitrogen, Carlsbad, CA, USA) digestion and phenol/chloroform method (34). The SNPs in RUNX2 binding site rs6086746, rs7179057 and rs1531268 were genotyped by iPLEX Gold SNP genotyping (35). We used an inter- and intra-replication validation to assess quality of genotyping experiment. Inter-replication validation was performed in 35 replicate samples (approximately 5%), and the concordance rate was 100%.

### 2.5 Ethical Statement

The study was reviewed and approved by the institutional ethics committee of the Tri-Service General Hospital (TSGH-2-102-05-028). After completely explaining the objectives of the study, written informed consent was obtained from all participants. All clinical and biological samples were collected, and DNA was genotyped after obtaining patient consent.

### 2.6 Luciferase Reporter Assays

The RUNX2 binding site SNP rs6086746 of PLCB4 luciferase reporter was amplified by polymerase chain reaction from the genomic DNA library of human immortalized myelogenous leukemia K562 cell with the primer pair: 5’: 5’-GGGGTACCCAGATACAAGCTACAACATGAATG-3’ and 3’: 5’-CCCAAGCTTCAATAAAGATATAAATCCTTTATAGCA-3’ and subcloned into a pGL3 basal reporter (Promega, USA) cut at KpnI and HidIII sites. After the sequence verification, we further changed the current A allele into G allele using the QuickChange Lightening Site-directed Mutagenesis Kit (Agilent Technology). The construction of pSG5.HA.RUNX2 (isoform 2) was performed by polymerase chain reaction from the K562 cell cDNA library with the primer pair: 5’: 5’-AACTCGAGGATGGCATCAAACAGCCTCTTCAGC-3’ and 3’: 5’-AAAGATCTTCAATATGGTCGCCAAACAGATTC-3’ and subcloned into a pSG5.HA vector (Stratagene, USA) cut at XhoI and BglII sites. HEK293 cells were cultured in Dulbecco’s modified Eagle’s medium supplemented with 10% charcoal/dextran-treated fetal bovine serum. The cells in each well (24-well plate) were transfected with total 1 μg DNA and jetPEI (PolyPlus-transfection, Illkirch, France) according to the manufacturer’s protocol. Luciferase activity was assessed after 24 h post transfection using the Promega Luciferase Assay Kit and expressed as mean relative light units (RLUs) of two transfected sets. Results shown are representative of at least three independent experiments.

### 2.7 RNA Extraction and qPCR Analysis

Total RNA was extracted from whole blood obtained from 5 osteoporosis participants, 7 osteopenia participants, and 7 controls by the TRIzol reagent method (Invitrogen, Carlsbad, CA) and then reverse transcribed into cDNA with the Script II 1st strand cDNA RT Kit (ACE biolabs, Taiwan). Real-time quantitative PCR (RT-qPCR) was performed to amplify cDNA with the 7500 fast Real-Time PCR System (Applied Biosystems) using the SYBR color qPCR Master Mix (ACE biolabs, Taiwan). Relative expression was analyzed by the comparative threshold cycle (Ct) method, and human glyceraldehyde-3-phosphate dehydrogenase (GAPDH) was used as an internal control. Expression values were calculated by the 2^–ΔCT^ method. The primer sequences used for genotyping of the SNPs and qPCR of RUNX2 and PLCB4 are shown in **Supplementary Table S1**. Melting curve analysis was used to confirm specificity, and three replicate wells were used for each subject.

### 2.8 Statistical Analysis

Continuous variables were evaluated using Student’s *t*-tests and reported as the mean ± S.D. Genotypes and allelic frequencies were compared between cases and controls using χ2 test or Fisher’s exact tests. Logistic regression was used to estimate ORs and 95% confidence intervals (CIs) as a measure of the association with osteopenia/osteoporosis susceptibility, adjusted by sex and age. The analysis was performed using allele type, genotype, dominant, and recessive models. Statistical analyses were performed using SPSS 22.0 (SPSS Inc., Chicago, Ill., USA) and R 3.5.2 (R Project for Statistical Computing, Vienna, Austria). A p-value of <0.05 was considered statistically significant.

## 3 Results

### 3.1 Selection of Candidate SNPs

**Figure 1:**
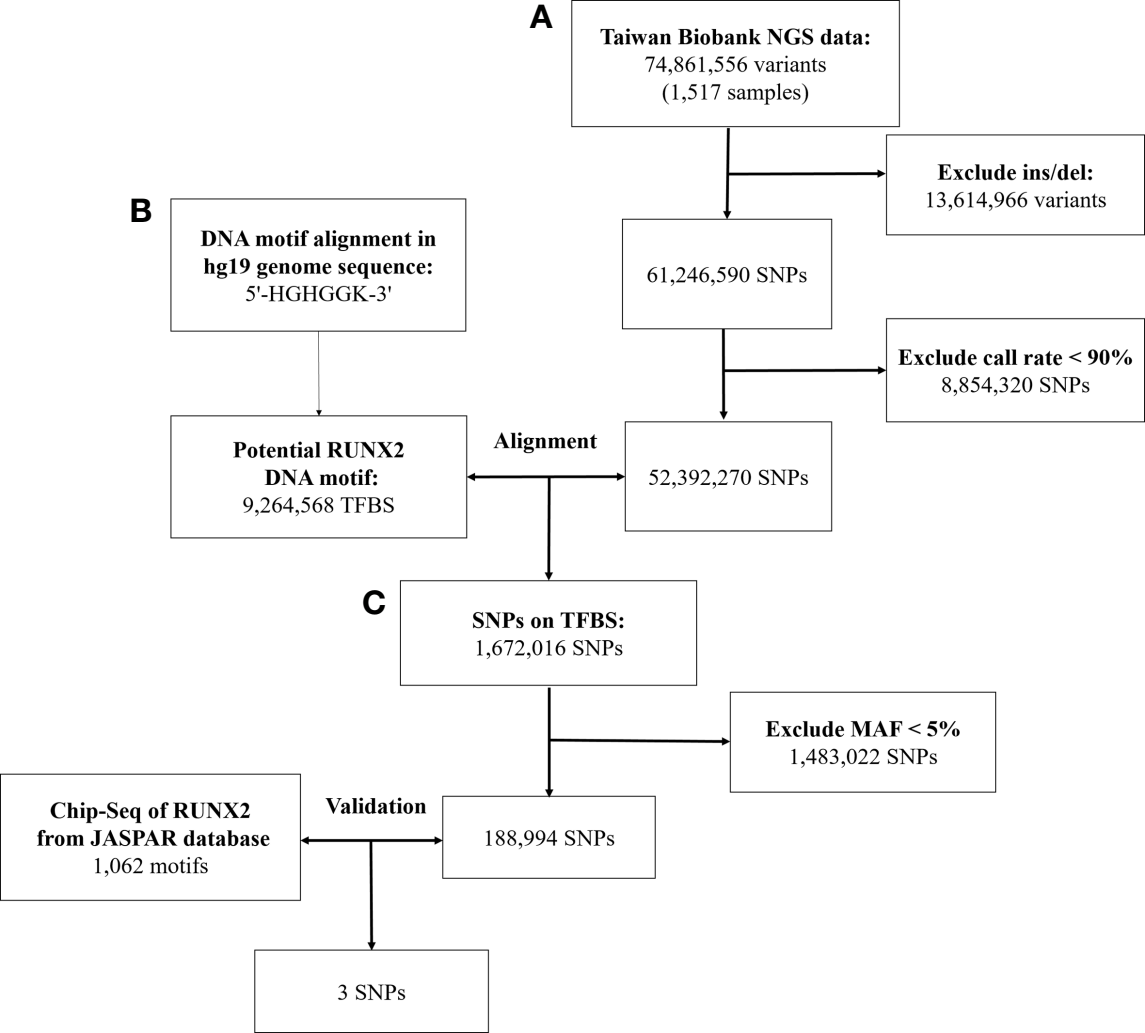
Workflow of the stepwise approach of candidate binding site SNPs. **(A)** Screen the genetic variation in the genome of Taiwanese through quality control procedures. **(B)** Identify genetic variants that may affect RUNX2 binding motif through bioinformatics sequence alignment. **(C)** Chromatin immunoprecipitation sequencing (ChIP-Seq) to confirm that these genetic variants will bind to these binding motifs. NGS, next-generation sequencing; SNP, single-nucleotide polymorphism; Ins/sel, insertion/deletion; TFBS, Transcription factor binding site; MAF, minor allele frequency.

In **Figure 1**, we used data from the NGS database of 1517 individuals from the Taiwan Biobank, which included 74,861,556 genetic variants. After excluding 13,614,966 structural variants (insertion/deletion) and 8,854,320 SNPs with a call rate of <90%, 52,392,270 SNPs were remaining.

With the human reference genome sequence (GRCh37/hg19) from the National Center for Biotechnology Information in combination with the Taiwan Biobank database. RUNX2 contained 9,264,568 potential binding motifs in the human genome based on the sequence of the above binding sites. Of the TFBSs, we compared the remaining 52,392,270 SNPs in the first step and found that 1,672,016 SNPs were in the TFBS of RUNX2. After excluding SNPs with minor allele frequency of <5%, 188,994 SNPs were remaining for further screening by ChIP-Seq analysis.

In the JASPAR database, there were 1062 positions in the TFBS associated with the RUNX2. After validating these SNPs with the results of the ChIP-Seq database of RUNX2, the remaining 3 SNPs are described in **Supplementary Table S2**. In the end, we used bioinformatics sequence alignment method and ChIP-Seq database for verification. Finally, 3 SNPs that may affect the binding ability of RUNX2 were screened to study the association with osteoporosis.

### 3.2 Population Characteristics

The basic clinical characteristics of the study population are summarized in **Table 1**. Osteoporosis and osteopenia patients had a lower weight than that the control subjects (p < 0.05). Osteoporosis and osteopenia patients had a lower BMI than the control subjects (p < 0.05).

**Table 1:** Characteristics of participants in case-control study.

	Control	Osteopenia	Osteoporosis	P-value
	(n = 254)	(n = 290)	(n = 107)	
Age (year)	72.95 ± 6.51	72.65 ± 6.43	73.36 ± 6.74	0.619
Age of menopause	49.84 ± 5.40	49.54 ± 4.82	48.77 ± 4.79	0.192
Height (cm)	154.46 ± 5.93	153.94 ± 5.48	153.21 ± 5.69	0.185
Weight (kg)	59.58 ± 9.04	56.36 ± 8.21	51.45 ± 7.63	<0.001*
BMI^a^	25.05 ± 3.74	23.96 ± 3.49	22.47 ± 3.16	<0.001*
BMD (g/cm^2^)	1.09 ± 0.15	0.89 ± 0.13	0.77 ± 0.16	<0.001*
T-score	0.11 ± 1.03	−1.73 ± 0.40	−2.96 ± 0.40	<0.001*
eGFR^b^	85.32 ± 21.07	86.61 ± 19.57	89.72 ± 20.94	0.194

*p-value < 0.05,

a: BMI, Weight/Height^2^,

b: eGFR, (MDRD-Simplify-GFR) (186* creatinine^-1.154^*age^-0.203^*0.742).

### 3.3 Association Between RUNX2 Binding Site Gene Polymorphisms With Susceptibility and Osteoporosis

Our results showed that SNP rs6086746 had a significant association with osteoporosis risk according to genotype (p < 0.001; **Table 2**). The minor allele frequency of rs1531268 (allele C), rs6086746 (allele A), and rs7179057 (allele A) in controls was 0.38, 0.21, and 0.13, respectively, which was similar to the frequency noted in Taiwan Biobank data. In the control group, the Hardy–Weinberg equilibrium p-values for rs1531268, rs6086746, and rs71790572 were 0.998, 0.160, and 0.157, respectively, which conforms to the Hardy–Weinberg equilibrium (p > 0.05).

**Table 2:** Genotypic characteristics of participants in this study.

	Control	Osteopenia	Osteoporosis	P-value
	(n=254)	(n=290)	(n=107)	
rs1531268				0.617
TT	97 (38.5%)	106 (36.9%)	46 (43.4%)	
TC	118 (46.8%)	134 (46.7%)	41 (38.7%)	
CC	37 (14.7%)	47 (16.4%)	19 (17.9%)	
rs6086746				<0.001*
GG	151 (60.4%)	156 (54.2%)	59 (55.1%)	
AG	93 (37.2%)	115 (39.9%)	32 (29.9%)	
AA	6 (2.4%)	17 (5.9%)	16 (15.0%)	
rs7179057				0.579
GG	192 (76.5%)	209 (72.8%)	84 (79.2%)	
AG	51 (20.3%)	71 (24.7%)	19 (17.9%)	
AA	8 (3.2%)	7 (2.4%)	3 (2.8%)	

*p-value < 0.05.

In **Table 3**, we used the logistic regression to compare the genotype and allele frequencies of psteopenia patients and control participants. Significant difference was found in the genotype model (GG vs. AA) in all the subjects with adjustment for age and BMI (OR = 3.56; 95% CI = 1.25–10.14; p = 0.017). Significant difference was found in the recessive model (GG+AG vs. AA) in all the subjects with adjustment for age and BMI (OR = 3.25; 95% CI = 1.15–9.15; p = 0.026). Moreover, a higher T allele frequency was associated with an increased risk of osteopenia (OR = 1.44; 95% CI = 1.05–1.98; p = 0.025). In **Table 4**, we used the logistic regression to compare the genotype and allele frequencies of osteoporosis patients and control participants. Significant difference was found in the genotype model (GG vs. AA) in all the subjects with adjustment for age and BMI (OR = 6.26; 95% CI = 1.99–19.68; p = 0.002). Significant difference was found in the recessive model (GG+AG vs. AA) in all the subjects with adjustment for age and BMI (OR = 6.89; 95% CI = 2.23–21.31; p = 0.001).

**Table 3:** Association of the rs6086746 with osteopenia.

Independent variable	Crude-OR (95% CI)	P-value	Adj-OR (95% CI)^#^	P-value
rs6086746				
GG	1		1	
AG	1.20 (0.84–1.70)	0.319	1.27 (0.85–1.88)	0.243
AA	2.74 (1.05–7.14)	0.039*	3.56 (1.25–10.14)	0.017*
Dominant model				
GG	1		1	
AG+AA	1.29 (0.92–1.82)	0.145	1.40 (0.96–2.06)	0.083
Recessive model				
GG+AG	1		1	
AA	2.55 (0.99–6.57)	0.053	3.25 (1.15–9.15)	0.026*
Allele model				
G	1		1	
A	1.31 (0.99–1.75)	0.061	1.44 (1.05–1.98)	0.025*

*p-value < 0.05; ^#^Adjust by age, BMI.

**Table 4:** Association of the rs6086746 with osteoporosis.

Independent variable	Crude-OR (95% CI)	P-value	Adj-OR (95% CI)^#^	P-value
rs6086746				
GG	1		1	
AG	0.88 (0.53–1.45)	0.62	0.75 (0.41–1.36)	0.339
AA	6.82 (2.55–18.28)	<0.001*	6.26 (1.99–19.68)	0.002*
Dominant model				
GG	1		1	
AG+AA	1.24 (0.79–1.96)	0.355	1.09 (0.63–1.87)	0.754
Recessive model				
GG+AG	1		1	
AA	7.15 (2.71–18.84)	<0.001*	6.89 (2.23–21.31)	0.001*
Allele model				
G	1		1	
A	1.61 (1.12 - 2.31)	0.011*	1.48 (0.96–2.28)	0.079

*p-value < 0.05; ^#^Adjust by age, BMI.

### 3.4 Associations Between rs6086746 SNP and BMD

**Figure 2:**
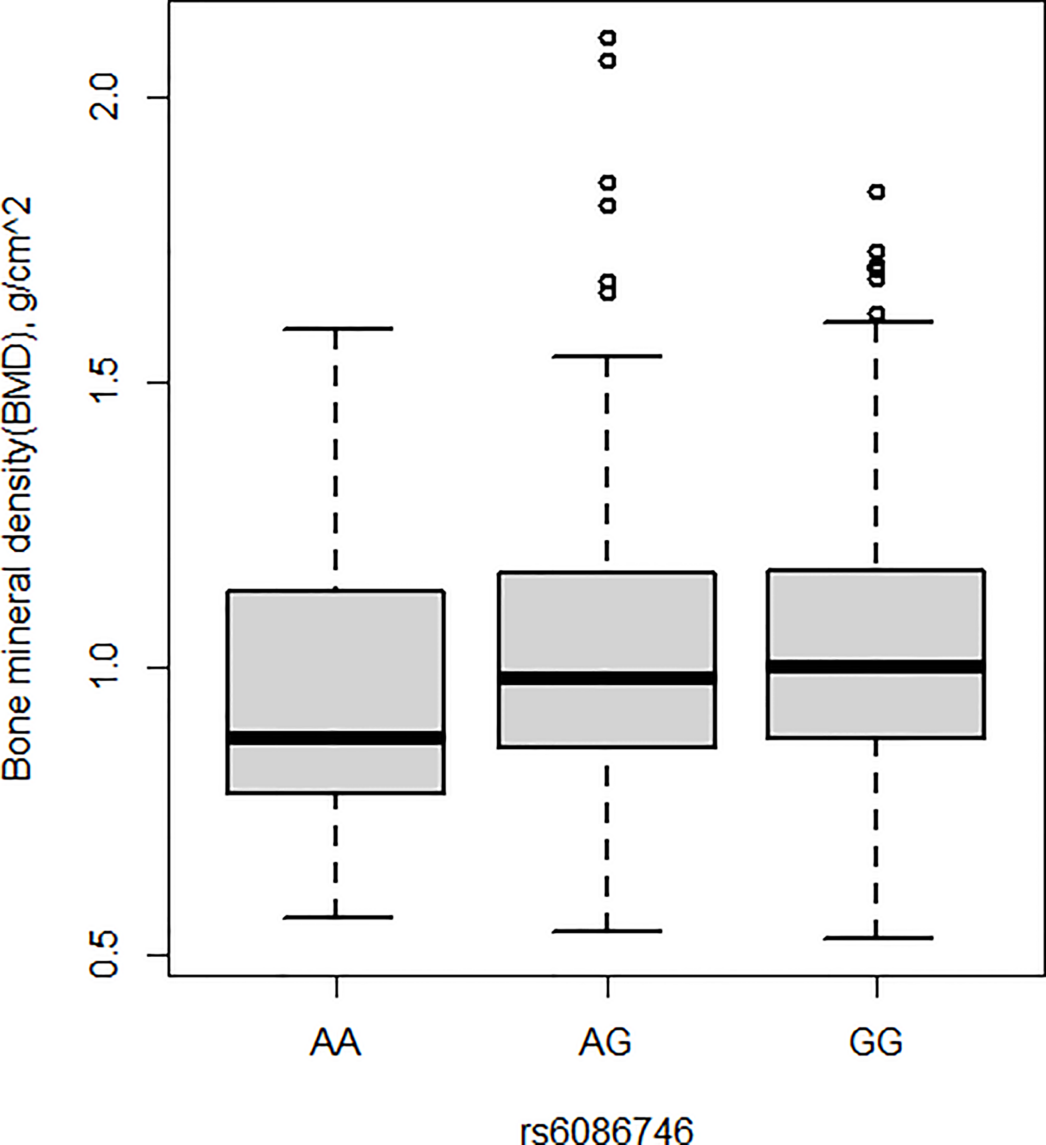
Association of bone mineral density and rs6086746 GG (n = 366), GA (n = 240), AA (n = 39). rs6086746 was determined by G or A in the position of -6363 relative to the PLCB4 gene. Numbers represent the population who has the specified genotype. BMD, expressed as an areal density in grams per square centimeter, was measured in the lumbar spine (L1–L4).

BMD was also measured in the current study. Patients with osteopenia/osteoporosis exhibited significantly lower total BMD and BMD of L1–L4 vertebrae compared with control subjects (p < 0.05, **Table 1**). Significant association was detected for the rs6086746 SNP with BMD levels (p < 0.05, **Figures 2**). However, individuals carrying the AA genotype at rs6086746 had significantly lower BMD levels (p < 0.05, **Figure 2**).

### 3.5 mRNA expression of RUNX2 and PLCB4 in whole blood

**Figure 3:**
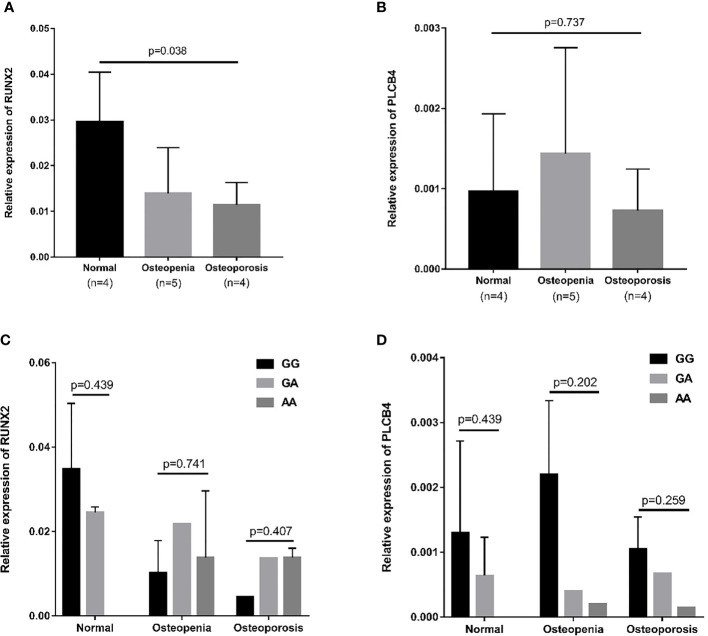
Relative mRNA expression in osteopenia, osteoporosis, and normal groups. **(A)** Relative mRNA expression of RUNX2: osteoporosis patients had lower mRNA expression than normal group (p = 0.008). **(B)** Relative mRNA expression of PLCB4: there is no difference in mRNA expression among the 3 groups (p = 0.737). The effect of the rs6086746 genotypes on the **(C)** RUNX2 and **(D)** PLCB4 mRNA expression level in the normal, osteopenia, and osteoporosis patient groups. There was no association between rs6086746 genotype and mRNA expression in each group. RUNX2, RUNX family transcription factor 2; PLCB4, phospholipase C beta 4.

The RUNX2 transcription factor binds to the promoter of PLCB4 to affect the expression of PLCB4 (**Figure 3**). Therefore, we examined the mRNA levels of RUNX2 and PLCB4 in whole blood extracted from 5 osteopenia patients, 4 osteoporosis patients and 4 controls. **Figure 3** shows the final number of experimental samples. Relative RUNX2 mRNA expression in whole blood was lower in osteoporosis patients than in controls (p = 0.038, **Figure 3A**). However, no difference in PLCB4 mRNA expression was found among the groups (p = 0.737, **Figure 3B**).

Next, we determined the expression of RUNX2 and PLCB4 in osteopenia/osteoporosis patients and controls with different rs6086746 alleles. In whole blood from controls or osteopenia/osteoporosis patients, no significant differences in RUNX2 and PLCB4 mRNA levels were found in any comparisons (**Figures 3C, D**). Experiments were performed in triplicates.

In addition, we separated the gene expression of different alleles and found that the gene with the G allele showed negative Pearson’s correlation coefficient: −0.283, p = 0.326 (**Figure S1A**); conversely, gene with the A allele showed positive Pearson’s correlation coefficient: −0.283, p=0.154 (**Figure S1B**). After testing, the Cohen’s q value of the correlation coefficient of the G and A alleles was 0.91, showing that there is a large difference between the two correlation coefficients. The A allele may change the affinity of RUNX2 and prevent it from binding, thereby causing RUNX2 to be unable to inhibit the promoter activity of rs6086746, which in turn increases PLCB4 expression.

### 3.6 Comparison of Promoter Activity of G and A Alleles of the PLCB4

**Figure 4:**
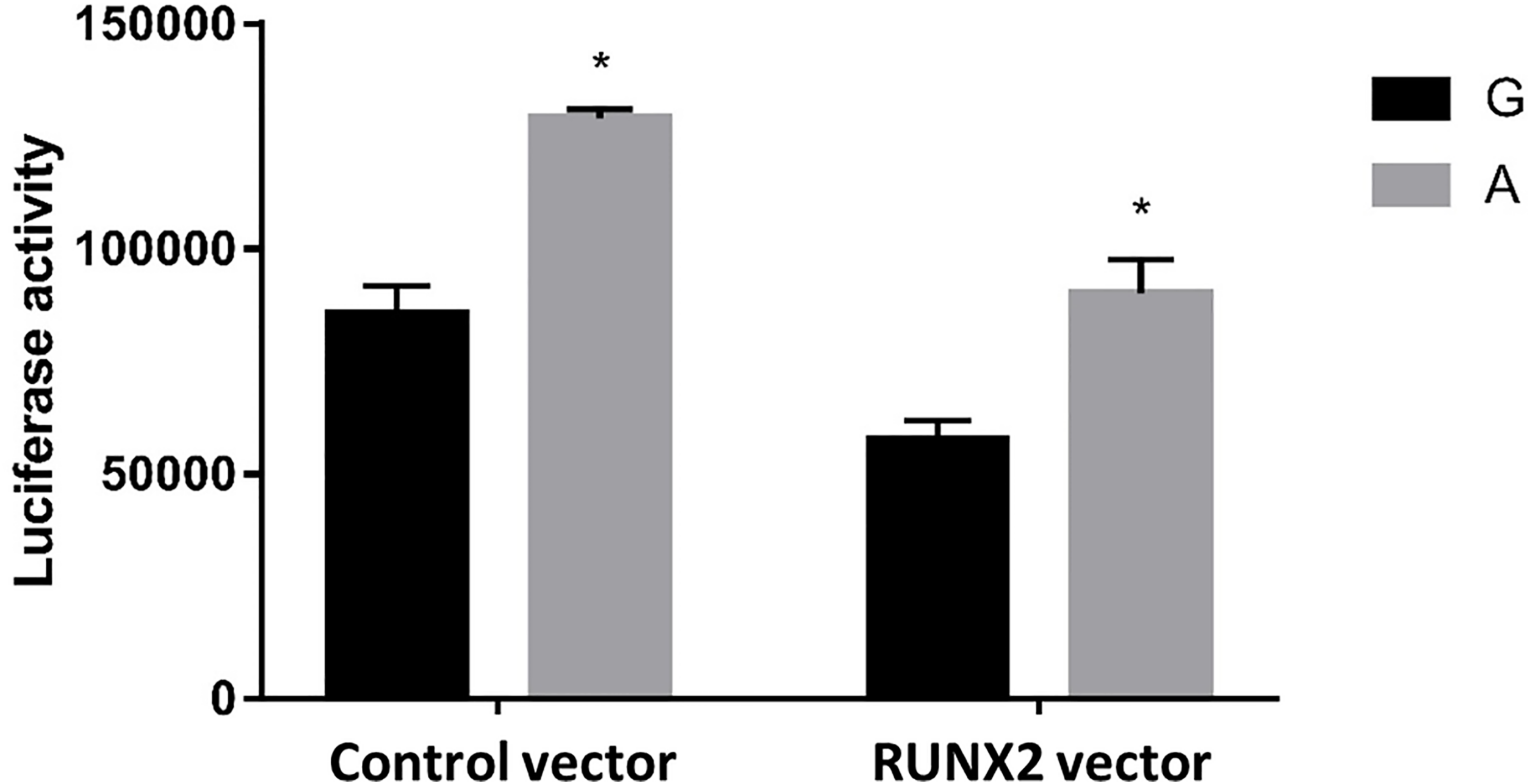
Effects of the rs6086746 genotype on luciferase activity in cultured HEK293cells. HEK293 cells were transfected with 0.5 μg pGL3 basic-LUC luciferase reporter recombinant plasmids containing a PLCB4 promoter sequence with the wild-type G allele or A allele at the rs6086746 SNP in the presence of 0.5 μg pSG5.HA control vector or pSG5.HA.RUNX2 vector. Transfected cells were cultured for 24 h. Luciferase activity in cell extracts was expressed in relative light units (RLUs). Mean ± SEM is given for each construct from three experiments. *p < 0.05.

To further test our hypothesis and to assess whether these enhancers cause allele-specific promoter activity, we cloned 268 bp regions containing individual allele of rs6086746. Indeed, our luciferase reporter assay data showed dramatic allelic difference of promoter activity. The promoter regions with the A allele (128975 ± 1979.87 RLU) at rs6086746 showed significantly higher activity to drive luciferase gene expression in HEK293 cells than those with the G allele (85478.67 ± 6281.75 RLU) (**Figure 4**). RUNX2 suppressed these promoter reporter activities. These results supported that these regions have allele-dependent enhancer activity, which is highly consistent with genotype-associated gene expression level (p < 0.05).

## 4 Discussion

In the current study, we investigated the association of SNPs in the RUNX2 binding site region with osteoporosis and showed that the rs6086746 polymorphism was significantly associated with osteoporosis. We also investigated the effect of SNPs on the expression level of PLCB4 in whole blood. In addition, luciferase activity of PLCB4 in individuals carrying the rs6086746 A allele was increased, suggesting a functional explanation for the observed association.

Because RUNX2 is a key molecule for osteoblast development, some studies have already been published regarding genomic association. Bustamante et al. (27) showed that the −1025 T/C polymorphism (rs7771980) in promoter 2 of RUNX2 is related to lumbar spine and femoral neck BMD in Spanish postmenopausal women. Auerkari et al. (36) showed that rs59983488 of RUNX2 promoter P1 region have been found to been associated with osteoporosis in postmenopausal Indonesian women. Qui et al. (37) suggested that osteoporosis GWAS-associated lead SNPs and their linked SNPs on the RUNX2 TF binding affinity. Previous studies all identified disease-related SNPs before identifying the transcription factor(s). Therefore, we provided an approach to use a hybrid method comprising candidate gene and epidemiologic approaches by first identifying specific disease-related transcription factors before identifying motif-binding regions.

SNP rs6086746 is located upstream of the PLCB4 gene, a large gene spanning 412 kb and containing 46 coding exons. The PLCB4 gene provides instructions for making one form (the beta 4 isoform) of a protein called phospholipase C. This protein is involved in a signaling pathway within cells known as the phosphoinositide cycle, which helps transmit information from outside the cell to inside the cell. Phospholipase C carries out one particular step in the phosphoinositide cycle: the conversion of a molecule called phosphatidylinositol 4,5-bisphosphate (PIP2) to two smaller molecules, inositol 1,4,5-trisphosphate (IP3) and 1,2-diacylglycerol. These smaller molecules relay messages to the cell that ultimately influence many cellular activities (38).

Study suggest that the beta 4 isoform of phospholipase C contributes to the development of the first and second pharyngeal arches (39). These embryonic structures ultimately develop into the jawbones, facial muscles, middle ear bones, ear canals, outer ears, and related tissues. This protein is also believed to play a role in vision, particularly in the function of the retina, which is a specialized tissue at the back of the eye that detects light and color. Diseases associated with PLCB4 include auriculocondylar syndrome 2 (40) and auriculo-condylar syndrome (41).

rs6140791 polymorphism of the PLCB4 and PLCB1 genes might be involved in the pathogenesis of coronary artery aneurysm in Kawasaki disease (42). In a GWAS study, it was demonstrated that the two genetic loci rs4794822 and rs2072910 (PSMD3–CSF3 locus in 17q21.1 and PLCB4 locus in 20p12) were significantly associated with the regulation of neutrophil count (43). However, to date, there has been no study that examined the correlation between PLCB4 and osteoporosis, and the related pathogenesis remains unclear. In this study, we found that the rs6086746 may affect the binding of the RUNX2 transcription factor to increase PLCB4 expression, thereby increasing the risk of osteoporosis.

In the blood mRNA expression experiment in our study, RUNX2 expression decreases as osteoporosis becomes more severe, which is similar to the results of previous studies (44). Moreover, animal experiments have confirmed that there is low RUNX2 expression in osteoporosis rats model (45). The expression of PLCB4 in blood is low. We searched the GTEx database and AceView (https://www.ncbi.nlm.nih.gov/ieb/research/acembly/av.cgi?db=human&term=PLCB4&submit=Go) and confirmed that PLCB4 gene expression levels in blood are low. Therefore, we recommend the examination of expression levels in other tissues in future studies. Although RNA expression in blood is low and there was no difference, we were unable to find rs60867462 mRNA expression data in the GTEx database. However, we were able to identify the SNP for rs60867462 D’=1 in linkage disequilibrium data, which proved that the expression level of PLCB4 increases as the number of minor alleles increases (**Supplementary Table S3**). Moreover, our reporter gene assay results show that the RUNX2-associated sequence has regulatory activity and that rs6086746 in this binding site is able to affect the binding of this sequence to RUNX2.

In this study, we employed a candidate method that was different from past studies. In our method, we examined the pathogenic contributions of gene mutations in the entire genome based on candidate gene linkage studies and GWAS. From our screening results, many novel SNPs were identified. This was followed by in-depth examination of the biological pathways that are affected by these SNPs and their correlation with diseases. However, only an extremely low number of causal variants were found to be directly related to disease in GWAS (46). Therefore, most of the SNPs found by GWAS are not causal variants and further fine-mapping is required (47). Hence, our whole genome screening method successfully identified 3 RUNX2-associated SNPs. In the future, multiple omics technologies, including genomics, transcriptomics, epigenomics, proteomics, and metabolomics, can be combined to identify the molecular factors contributing to the pathogenesis and thereby address genetic susceptibility to disease development.

Certain potential limitations of this study may have influenced the results, such as the participants in this study were Asians; hence, the inferences may not be generalized to other populations. Secondly, regarding mRNA expression level, we were only able to use whole blood for gene expression experiments because most osteoporosis patients did not undergo invasive treatment. This resulted in very low PLCB4 expression. Third, the etiology of osteoporosis is relatively complex and is jointly affected by several genes. Therefore, the effect of a single gene or a single-nucleotide polymorphism on osteoporosis may be lower. We recommend that the effects of different transcription factors on osteoporosis be examined in the future, which may provide novel information on the genetic background underlying osteoporosis.

## 5 Conclusions

**Figure 5:**
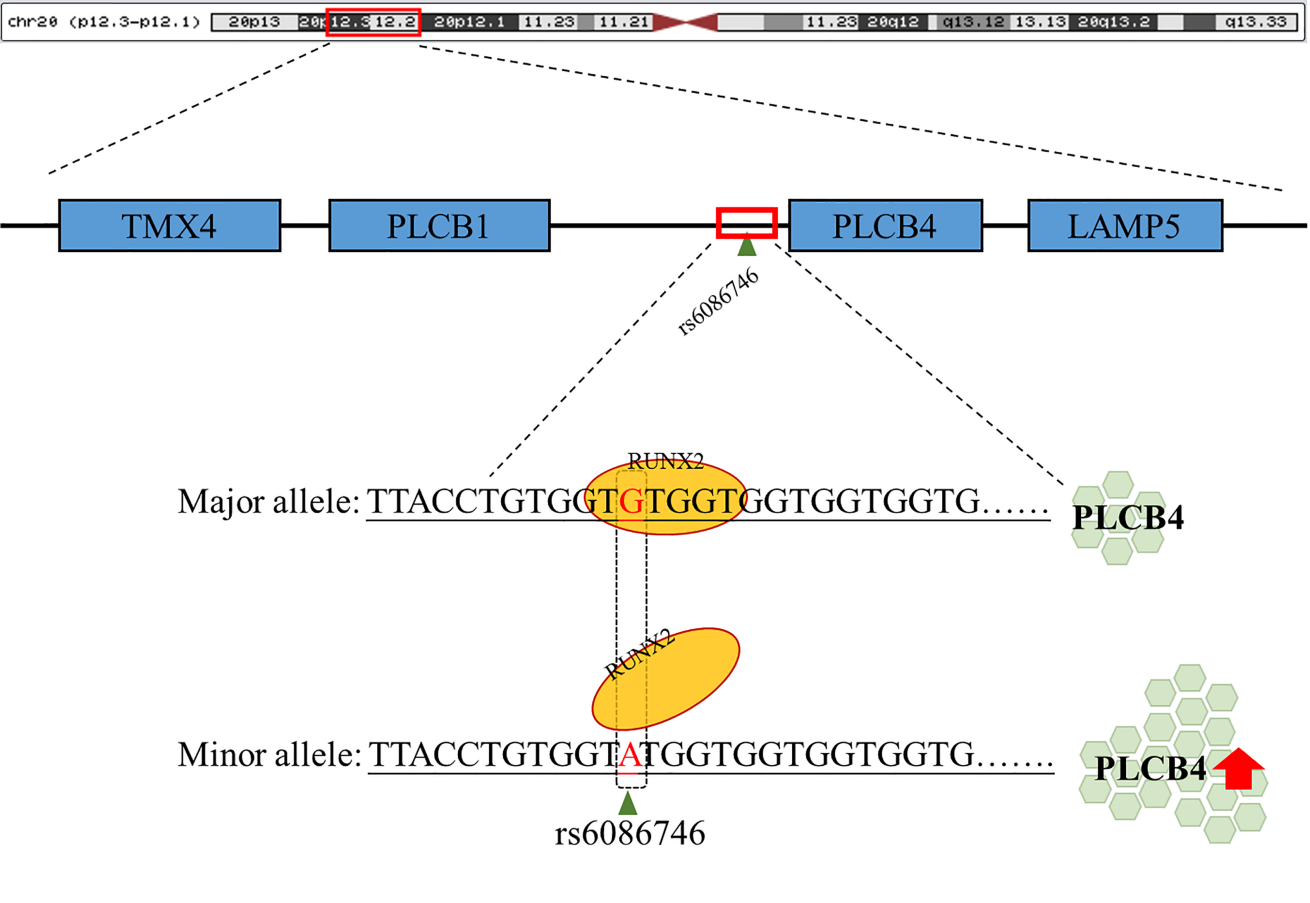
Schematic diagram of the binding of RUNX2 transcription factor to the PLCB4 promoter region. rs6086746 is located in the promoter region of PLCB4, and when G allele is mutated to A allele, it may affect the binding ability of runx2 transcription factors. This prevents the RUNX2 transcription factor from binding to its binding motif, thereby increasing the expression level of the downstream PLCB4 gene.

In summary, our data demonstrated that rs6086746 plays an important role in postmenopausal women with osteoporosis susceptibility, modulating the epigenetic regulation of a critical osteoporosis-related gene, PLCB4. rs6086746 impairs the binding of RUNX2 to the promoter of PLCB4, which may lead to enhanced expression of the PLCB4 gene(**Figure 5**). However, the impact of the RUNX2 binding site SNP rs6086746 and PLCB4 on the development and function of osteoporosis remains incompletely understood, and further exploration of the regulatory mechanism is warranted.

## Data Availability Statement

The datasets presented in this study can be found in online repositories. The names of the repository/repositories and accession number(s) can be found below: European Variation Archive [accession: PRJEB47913].

## Ethics Statement

The study was reviewed and approved by the institutional ethics committee of the Tri-Service General Hospital (TSGH-2-102-05-028). The patients/participants provided their written informed consent to participate in this study.

## Author Contributions

D-JT, W-HF, and S-LS: Conception and design. D-JT, LW-W, M-CT, C-CK, P-JH, C-CC, and WS: Acquisition of data. D-JT, S-MH, W-TC, and C-CW: Data analysis. D-JT: Drafting the article. D-JT, W-HF, LW-W, M-CT, C-CK, S-MH, W-TC, P-JH, C-CC, WS, C-CW, and S-LS have made substantial contributions in the interpretation of data, revising the article critically, and all approved of the final version for submission. All authors contributed to the article and approved the submitted version.

## Funding

This study was supported by grants from the Tri-Service General Hospital (TSGH-E-110231, TSGH-E-110232), Ministry of Science and Technology (MOST107-2314-B016-052-MY3, MOST110-2314-B016-006), Taoyuan Armed Forces General Hospital (TYAFGH-D-109009, TYAFGH-E-109052, TYAFGH-D-110032, TYAFGH-A110023), National Defense Medical Center(MND-MAB-110-105), Cheng Hsin General Hospital (CHNDMC-109-8, CHNDMC-110-16).

## Conflict of Interest

The authors declare that the research was conducted in the absence of any commercial or financial relationships that could be construed as a potential conflict of interest.

## Publisher’s Note

All claims expressed in this article are solely those of the authors and do not necessarily represent those of their affiliated organizations, or those of the publisher, the editors and the reviewers. Any product that may be evaluated in this article, or claim that may be made by its manufacturer, is not guaranteed or endorsed by the publisher.

## Supplementary Material

Supplementary Figure 1 Scatter plot of RUNX2 and PLCB4 expression levels in whole blood. **(A)** Scatter plot of all the expressed genes in G allele. The red line demonstrates negative correlation between RUNX2 and PLCB4 with Pearson’s correlation coefficient of 0.283. **(B)** Scatter plot of all the expressed genes in A allele. The blue line demonstrates positive correlation between RUNX2 and PLCB4 with Pearson’s correlation coefficient of 0.554. After the correlation coefficients of the two groups were tested, Cohen’s q value was 0.92. Runt-related transcription factor 2 (RUNX2; y-axis) versus phospholipase C beta 4 (PLCB4; x-axis) mRNA expression value. Cohen’s q value is used to compare the effect size of the differences between the two correlation coefficients (r). A q value of 0.1 means that there is almost no difference, a q value of 0.3 means moderate difference, and a q value of 0.5 means that there is a large difference.
