# Supplementary figures and images for: The Polymorphism at PLCB4 Promoter (rs6086746) Changes the Binding Affinity of RUNX2 and Affects Osteoporosis Susceptibility: An Analysis of Bioinformatics-Based Case-Control Study and Functional Validation
Source: Front Endocrinol (Lausanne). 2021 Nov 25;12:730686. doi: 10.3389/fendo.2021.730686 (PMC8657146; doi:10.3389/fendo.2021.730686)

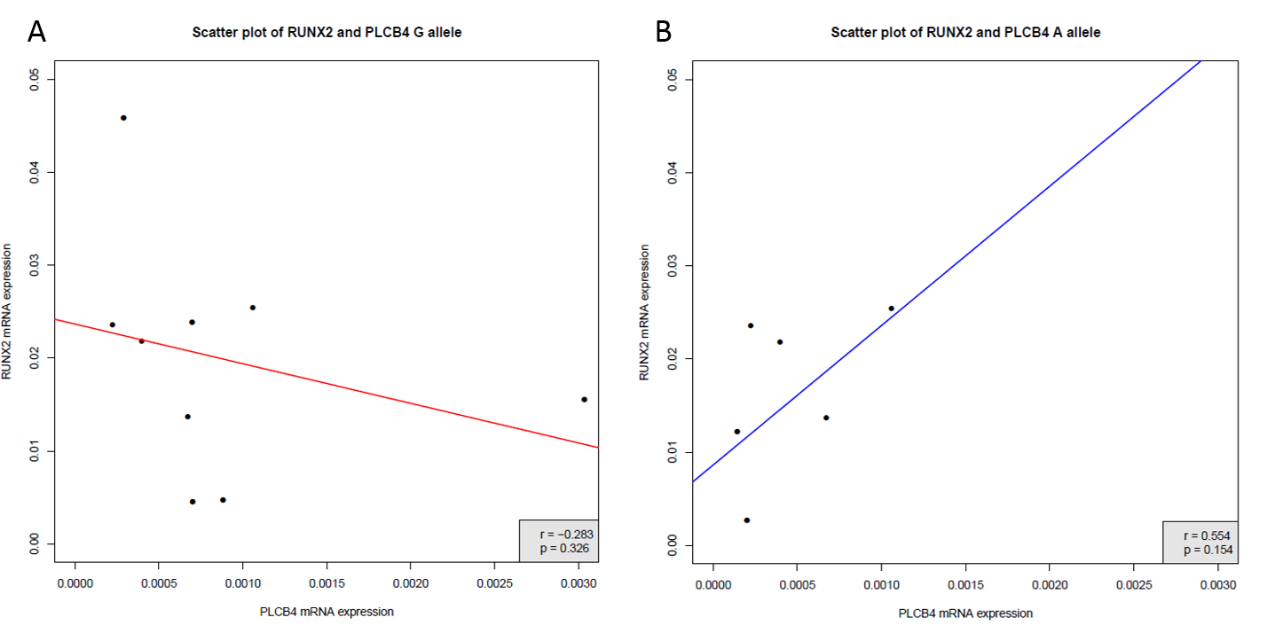

Supplement: Supplementary Figure 1 — Scatter plot of RUNX2 and PLCB4 expression levels in whole blood. (A) Scatter plot of all the expressed genes in G allele. The red line demonstrates negative correlation between RUNX2 and PLCB4 with Pearson’s correlation coefficient of 0.283. (B) Scatter plot of all the expressed genes in A allele. The blue line demonstrates positive correlation between RUNX2 and PLCB4 with Pearson’s correlation coefficient of 0.554. After the correlation coefficients of the two groups were tested, Cohen’s q value was 0.92. Runt-related transcription factor 2 (RUNX2; y-axis) versus phospholipase C beta 4 (PLCB4; x-axis) mRNA expression value. Cohen’s q value is used to compare the effect size of the differences between the two correlation coefficients (r). A q value of 0.1 means that there is almost no difference, a q value of 0.3 means moderate difference, and a q value of 0.5 means that there is a large difference. [file Image_1.tif]
