# Supplementary material for: The Polymorphism at PLCB4 Promoter (rs6086746) Changes the Binding Affinity of RUNX2 and Affects Osteoporosis Susceptibility: An Analysis of Bioinformatics-Based Case-Control Study and Functional Validation
Source: Front Endocrinol (Lausanne). 2021 Nov 25;12:730686. doi: 10.3389/fendo.2021.730686 (PMC8657146; doi:10.3389/fendo.2021.730686)
Supplement: Supplementary file 2 [file Table_1.docx]

Supplemental Table 1: Sequences of qPCR primers

| Gene | Forward primer | Reverse primer |
| --- | --- | --- |
| GAPDH | AGGTCGGAGTCAACGGATTT | ATCTCGCTCCTGGAAGATGG |
| RUNX2 | GGCAATGACGAGAACTACTCCG | GATGGTCAGGGTGAAACTCTTCC |
| PLCB4 | CAGGCAAAGTGACAGGAAAAG | CAGGTGATGTCCACTTCGTC |
