# Supplementary material for: The Polymorphism at PLCB4 Promoter (rs6086746) Changes the Binding Affinity of RUNX2 and Affects Osteoporosis Susceptibility: An Analysis of Bioinformatics-Based Case-Control Study and Functional Validation
Source: Front Endocrinol (Lausanne). 2021 Nov 25;12:730686. doi: 10.3389/fendo.2021.730686 (PMC8657146; doi:10.3389/fendo.2021.730686)
Supplement: Supplementary file 3 [file Table_2.docx]

Supplemental Table 2. Summary of three candidate SNPs obtained from bioinformatics analyses

| Chromosome | position | SNP ID | MAF | DNA sequence near the SNP | Gene |
| --- | --- | --- | --- | --- | --- |
| 15: | 102390506 | rs7179057 | 12.40%(G→A) | TTGTG[G/A]TCATG | OR4F13P |
| 17: | 73805613 | rs1531268 | 39.24%(T→C) | CGAGG[T/C]CCCTG | UNK |
| 20: | 9042994 | rs6086746 | 25.94%(G→A) | GTGGT[G/A]TGGTG | PLCB4 |
