# Supplementary material for: The Polymorphism at PLCB4 Promoter (rs6086746) Changes the Binding Affinity of RUNX2 and Affects Osteoporosis Susceptibility: An Analysis of Bioinformatics-Based Case-Control Study and Functional Validation
Source: Front Endocrinol (Lausanne). 2021 Nov 25;12:730686. doi: 10.3389/fendo.2021.730686 (PMC8657146; doi:10.3389/fendo.2021.730686)
Supplement: Supplementary file 4 [file Table_3.docx]

| Supplemental Table 3: 10 SNPs with D’=1 near rs6086746 and their expressions quantitative trait loci analysis (eQTL) from the GETx-Portal | | | |
| --- | --- | --- | --- |
| SNP | eQTL violin plot | NES | P-Value |
| rs6108249 | 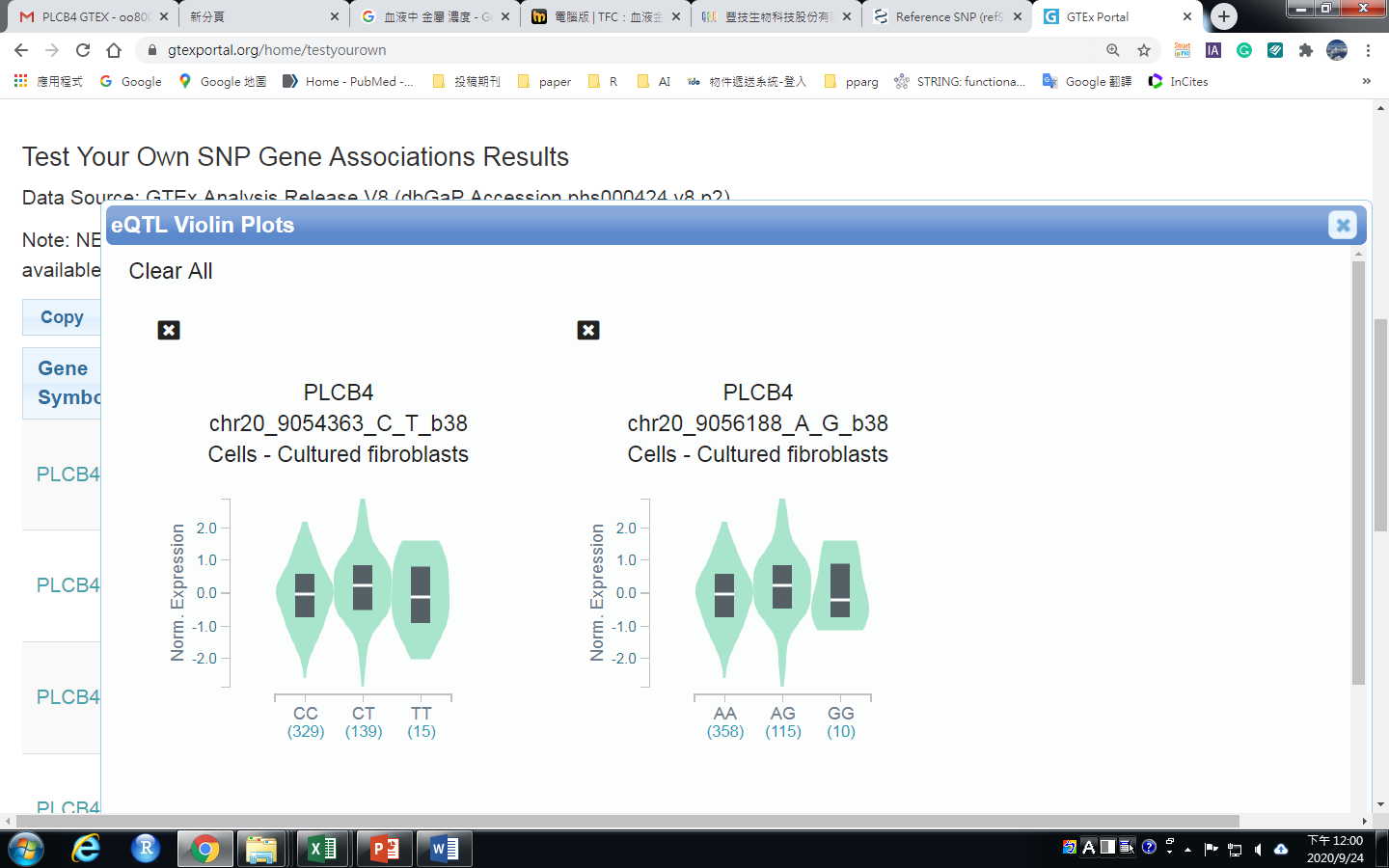 | 0.35 | 2.70E-09 |
| rs11700126 | 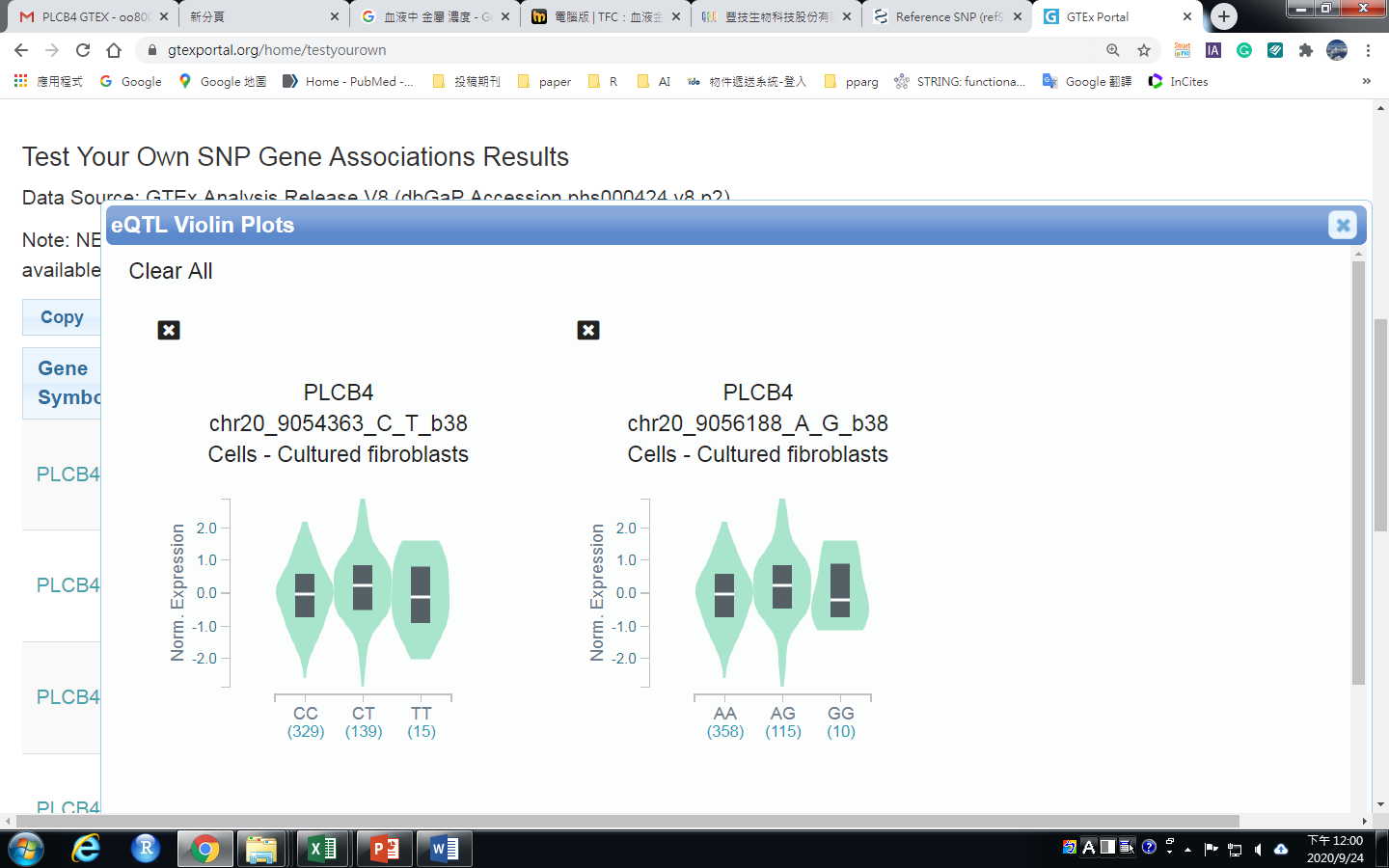 | 0.42 | 1.10E-11 |
| rs16995506 | 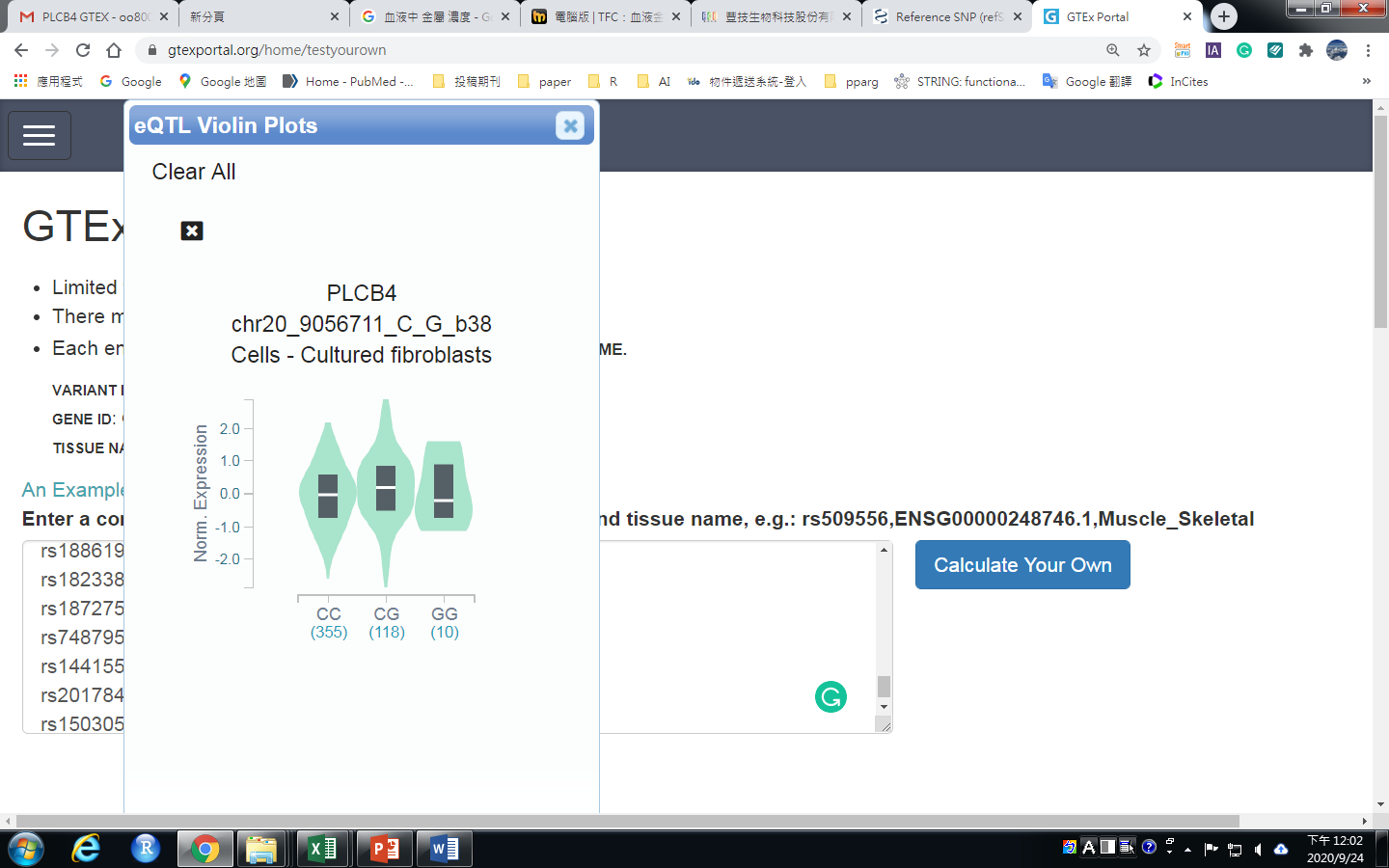 | 0.4 | 5.80E-11 |
| rs6039369 | 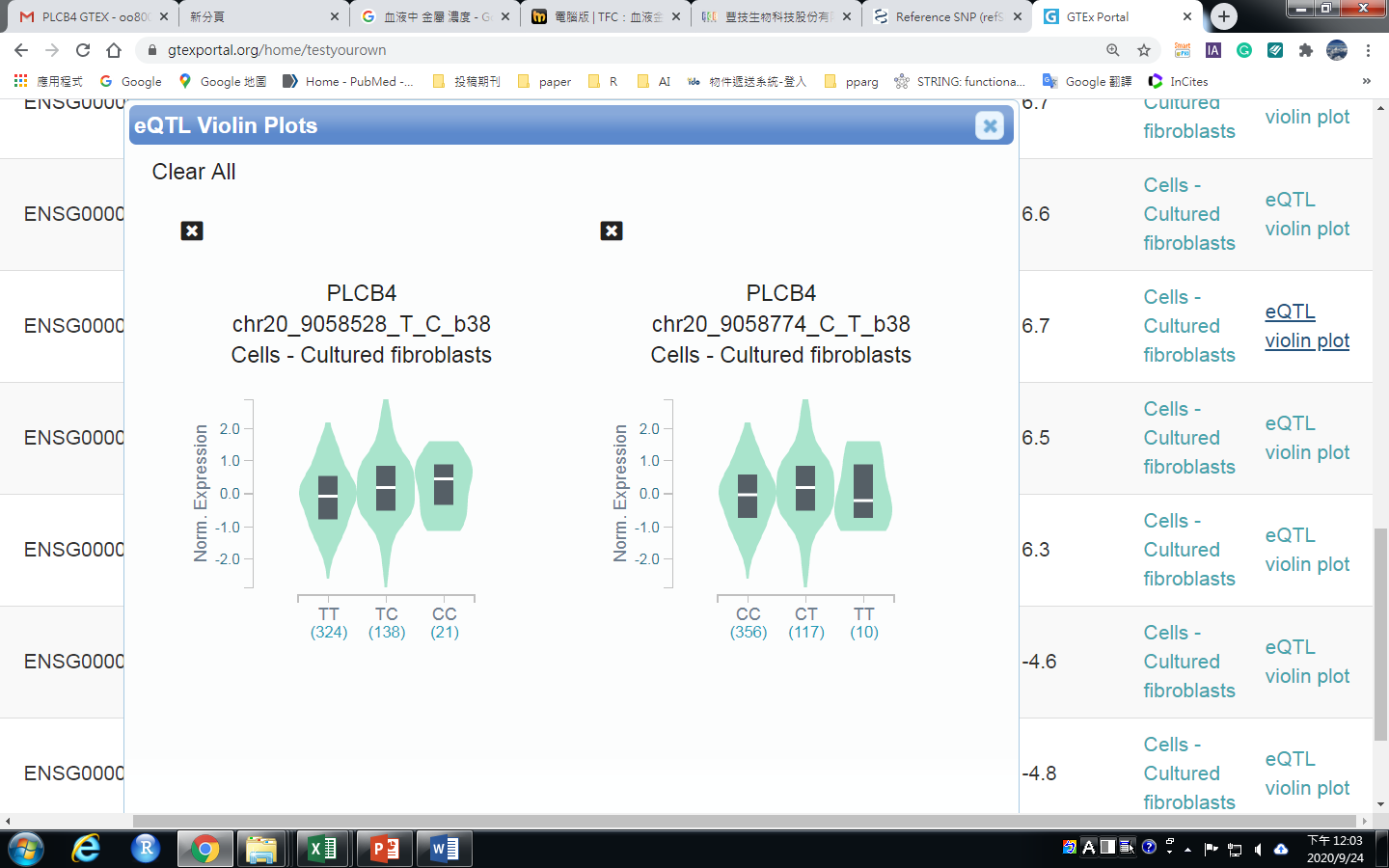 | 0.38 | 1.60E-10 |
| rs6056369 | 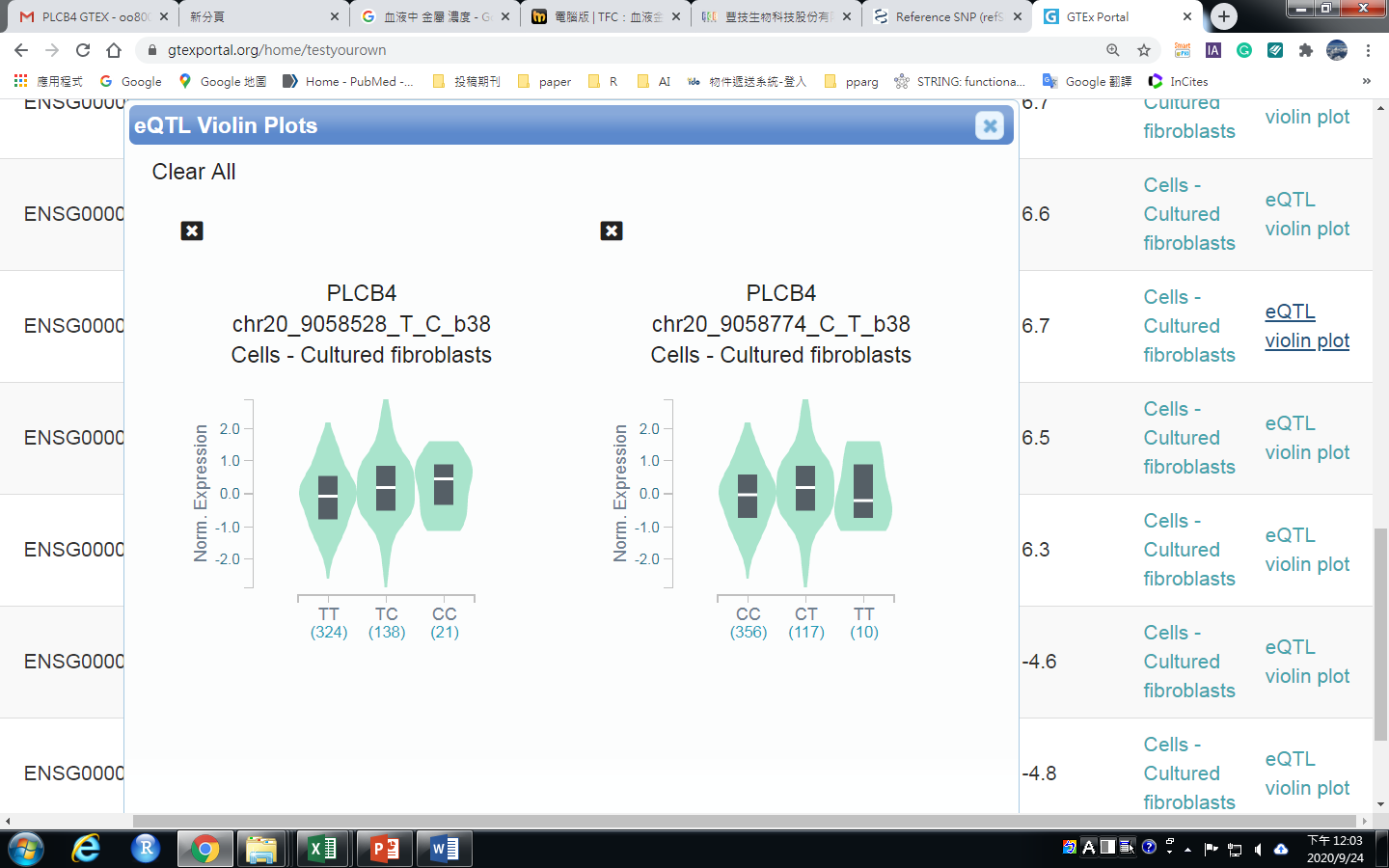 | 0.4 | 8.60E-11 |
| rs6056373 | 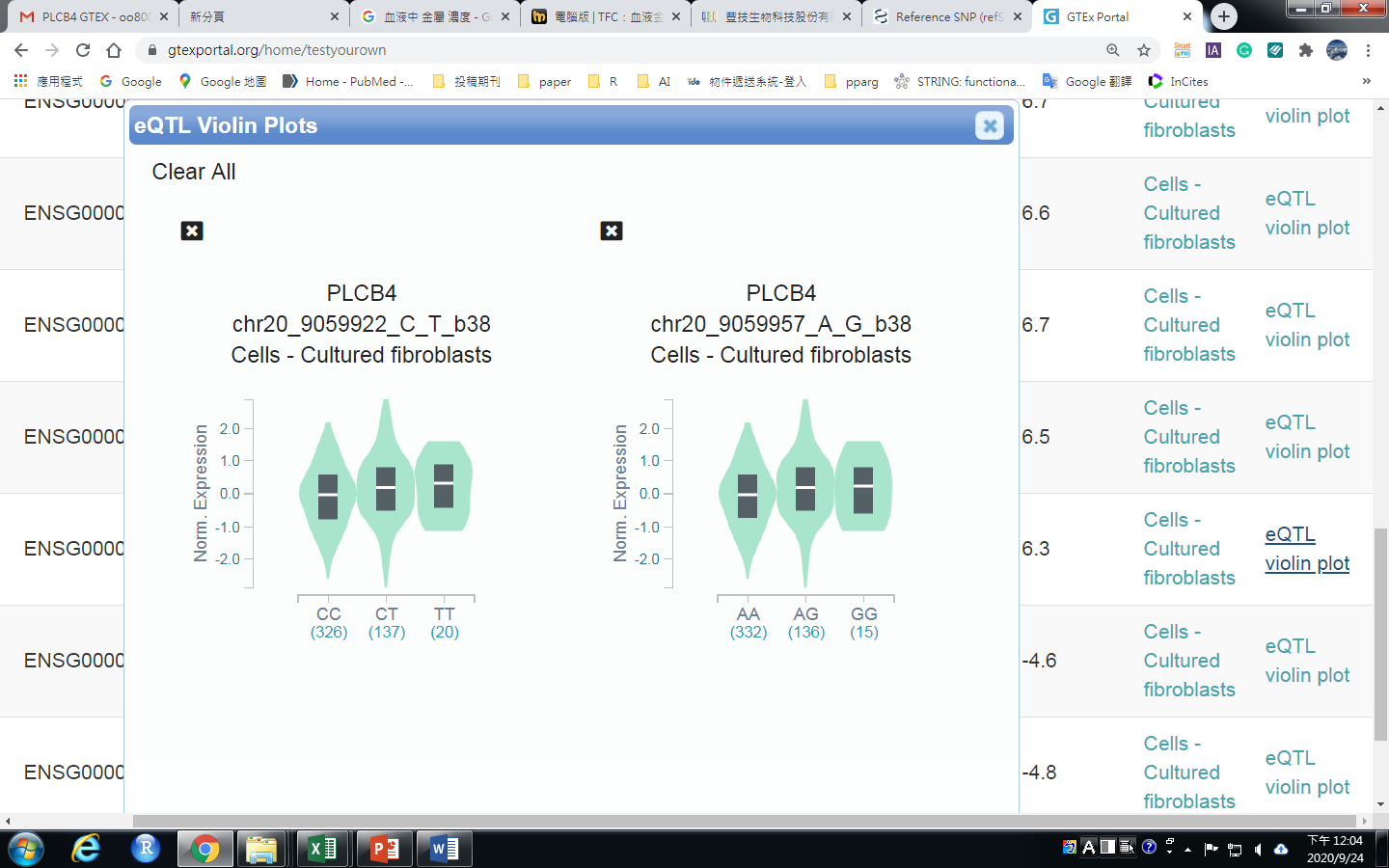 | 0.38 | 2.10E-10 |
| rs6056374 | 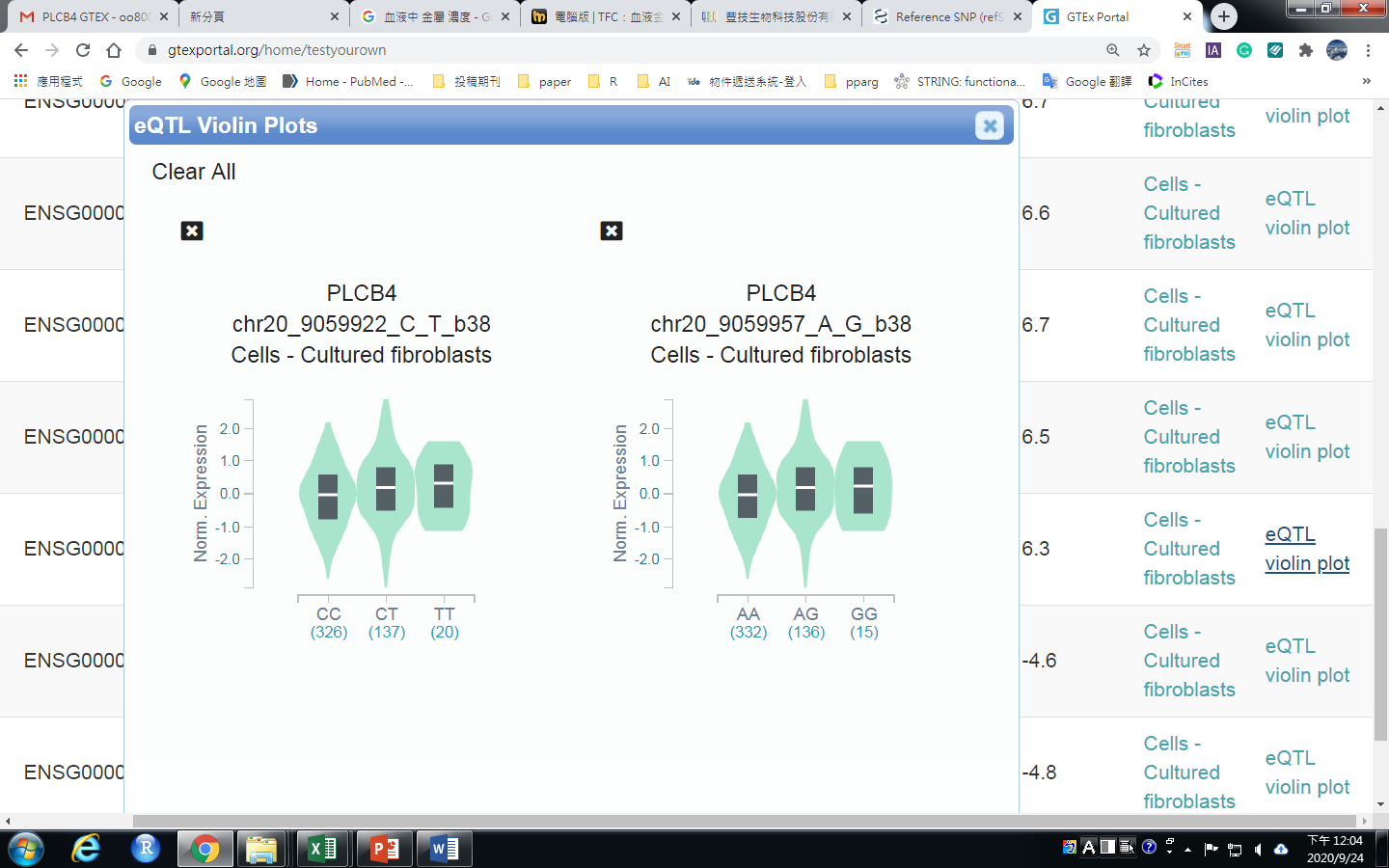 | 0.36 | 9.20E-10 |
| rs6086747 | 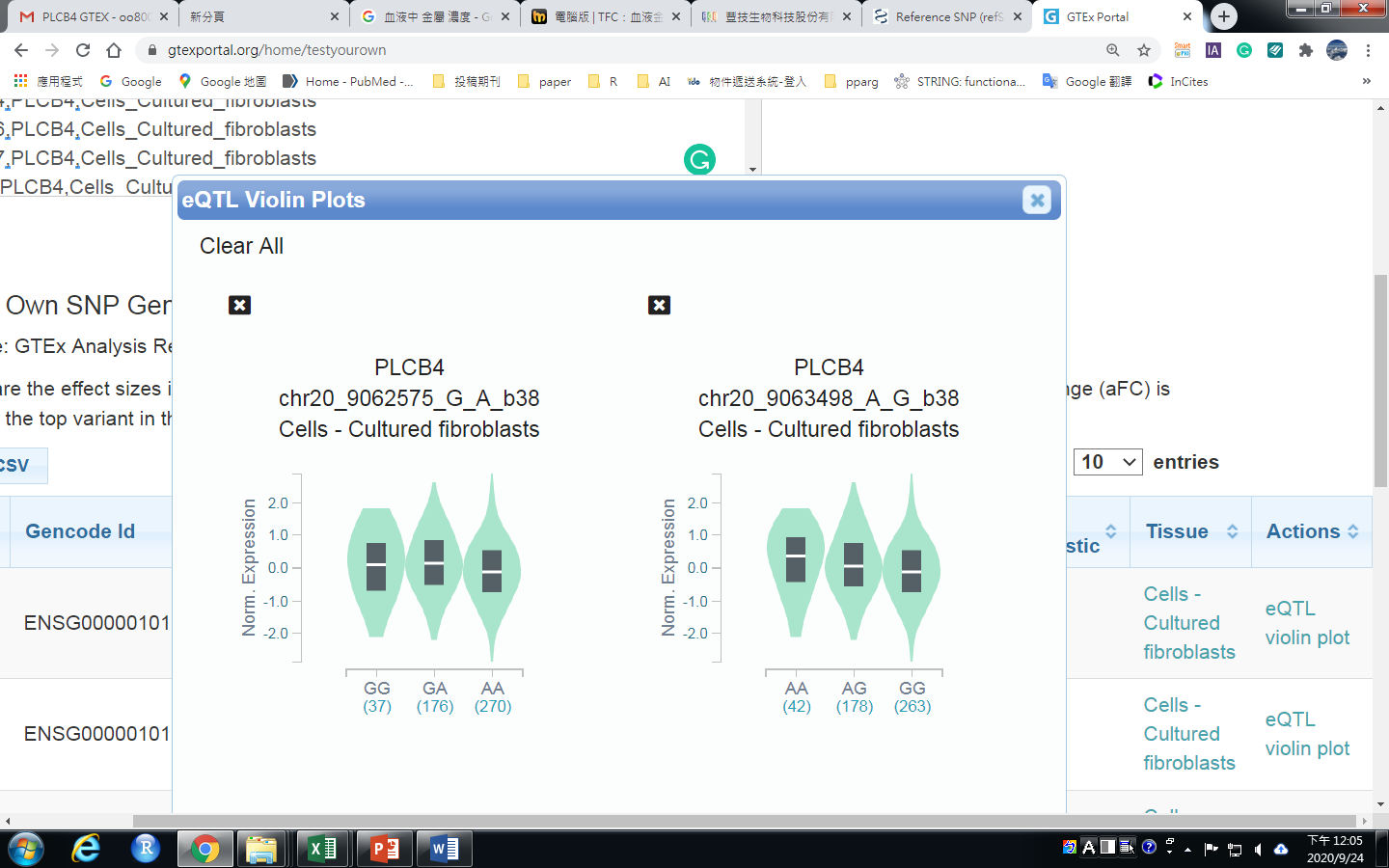 | -0.22 | 0.0000069 |
| rs927155 | 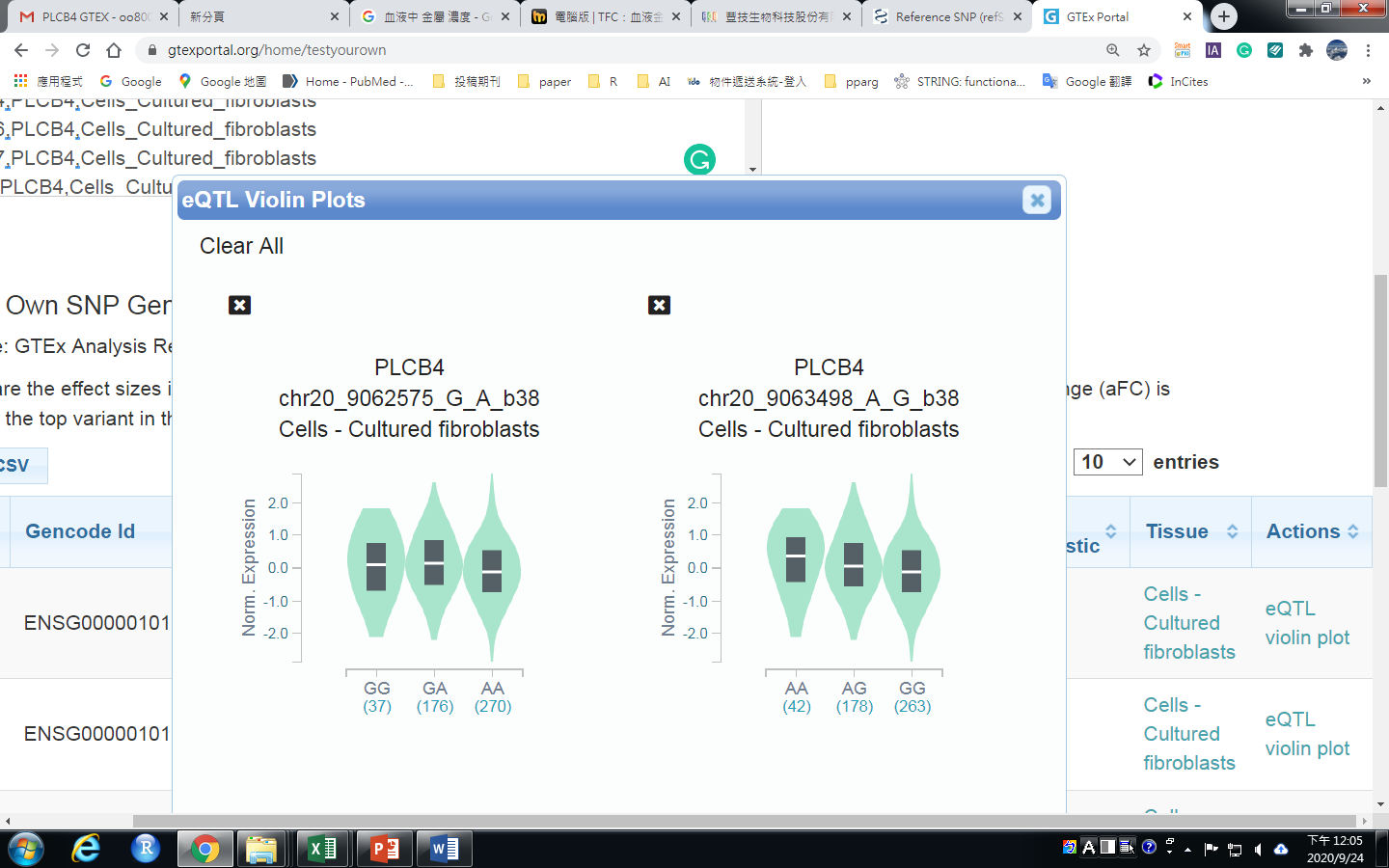 | -0.23 | 0.0000027 |
| rs6056378 | 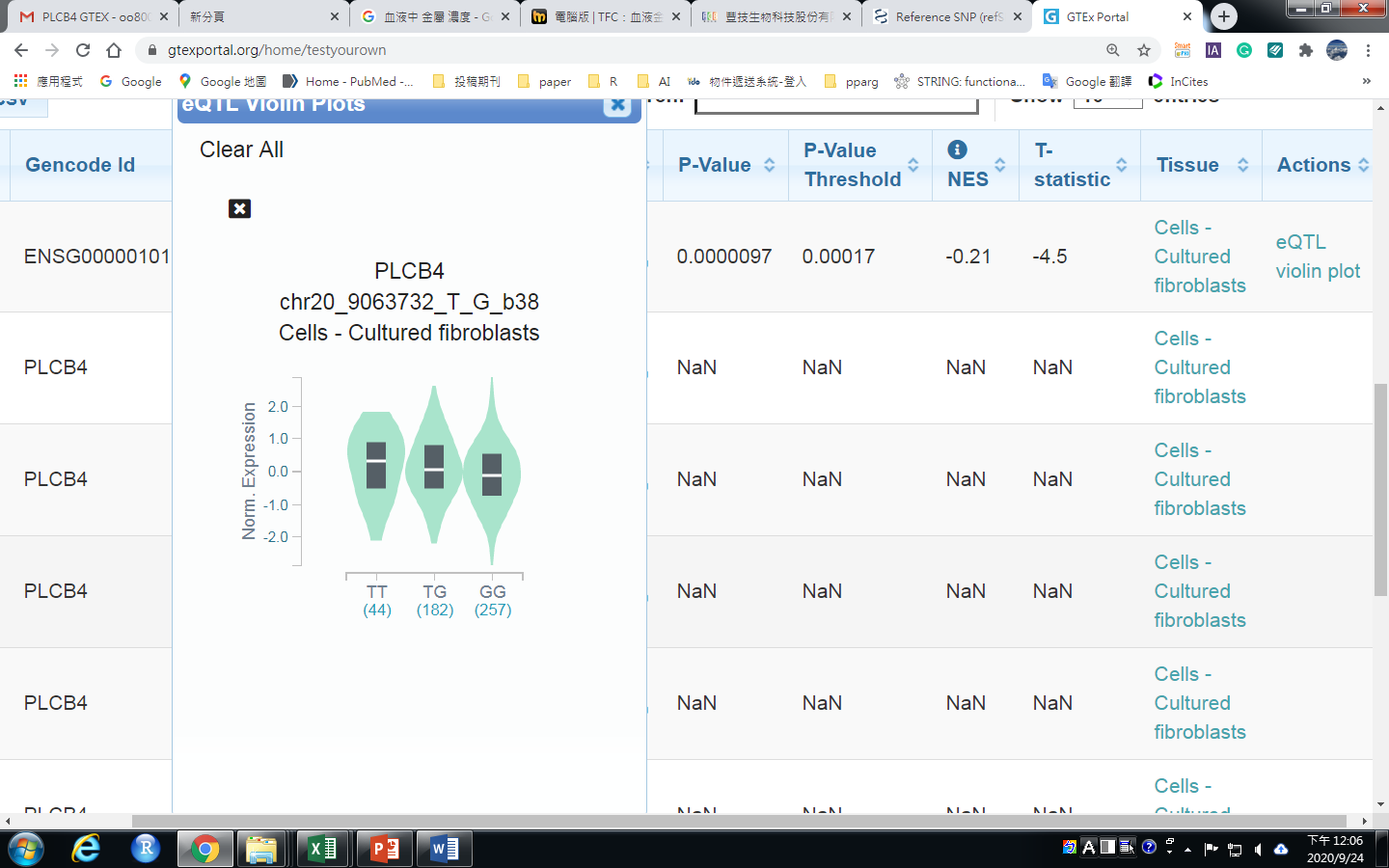 | -0.21 | 0.0000097 |
